# High expression of PD-L1 mainly occurs in non-small cell lung cancer patients with squamous cell carcinoma or poor differentiation

**DOI:** 10.32604/or.2023.028227

**Published:** 2023-05-24

**Authors:** LU LIU, BIN XIE, WEI ZHU, QIUYAN HE, JIANHUA ZHOU, SHUANG LIU, YONGGUANG TAO, DESHENG XIAO

**Affiliations:** 1 Department of Pathology, Xiangya Hospital, Central South University, Changsha, China; 2 Department of Pathology, School of Basic Medicine, Central South University, Changsha, China; 3 Center for Medicine Research, Xiangya Hospital, Central South University, Changsha, China; 4 Key Laboratory of Carcinogenesis and Cancer Invasion (Central South University), Ministry of Education, Cancer Research Institute, School of Basic Medicine, Central South University, Changsha, China

**Keywords:** NSCLC, PD-L1, Immunotherapy, LUSC, LUAD

## Abstract

**Background:**

Lung cancer is one of the most lethal cancers worldwide, but studies have shown that the higher the expression of programmed cell death protein 1 ligand 1 (PD-L1) in non-small cell lung cancer (NSCLC), the more likely it will benefit from anti-PD-L1 immunotherapy. The purpose of our study was to collect and analyze abundant clinical samples in order to provide evidence for clinicians and patients who might consider anti-PD-L1 immunotherapy while jointly formulating treatment plans.

**Methods:**

On the one hand, we obtained cases from The Cancer Genome Atlas (TCGA) database, including 498 lung squamous cell cancer (LUSC) patients and 515 lung adenocarcinoma (LUAD) patients. We studied the lung caner driver gene in LUSC and LUAD. On the other hand, PD-L1 expression was detected in lung cancer tissues of 1,008 NSCLC patients with immunohistochemistry staining (IHC), and we studied the correlation between PD-L1 protein expression and clinicopathological characteristics.

**Results:**

PD-L1 expression was higher in LUSC than in LUAD at the mRNA level. In univariate analysis, PD-L1 expression at the protein level was higher in patients who were males, were LUSC, were smokers, had a tumor diameter >3 cm, had poor differentiation, or had stages III~IV disease. In multivariate analysis, PD-L1 expression was higher in patients who were LUSC or in poor differentiation.

**Conclusion:**

In term of protein level, PD-L1 expression was higher in NSCLC patients who were LUSC or in poor differentiation. We recommend that PD-L1 IHC detection can be routinely performed in such populations that are likely to benefit most from PD-L1 immunotherapy.

## Introduction

Lung cancer is one of the major causes of cancer deaths worldwide. It is estimated that over the disease has killed more than 1.76 million individuals annually in recent years [1]. There are no obvious symptoms in early-stage lung cancer, thus the disease is typically discovered in the advanced stage. Hence, diagnosis and treatment play significant roles in all research on lung cancer. With the exception of hematoxylin and eosin (HE) staining, immunohistochemical (IHC) staining and even genetic testing are gradually being used for better diagnosis of lung cancer. More precise tumor classification makes it more likely for patients to obtain better prognoses and accurate individual therapy.

Lung cancer has traditionally been divided into small cell lung cancer (SCLC) and non-small cell lung cancer (NSCLC), with the former accounting for approximately 20% and the latter accounting for approximately 80% of all cases. The main subtypes of NSCLC include lung adenocarcinoma (LUAD), lung squamous cell cancer (LUSC), and large cell cancer (LCC) [2]. With great developments in molecular biology and precision medicine, the treatment of non-small cell lung cancer patients has changed from traditional surgery, radiotherapy, and chemotherapy to molecular-targeted therapy and immunotherapy. Due to the discovery of biomarkers and driver genes, a growing number of drugs for targeted therapy and immunotherapy have been approved by the Food and Drug Administration (FDA). Three of them have been listed in China and are aimed at Programmed cell death protein 1 ligand 1 (PD-L1), Epidermal growth factor receptor (*EGFR*), or Anaplastic lymphoma kinase (*ALK*) [3–5]. Compared with *EGFR* and *ALK*, PD-L1 is relatively novel; arguably, the advent of anti-programmed death cell protein-1 (PD-1) and anti PD-L1 are the most important innovations in the past decade; these innovations have proven to evade the immune system and promote remarkable antitumor activity in NSCLC [4,6]. Naturally, many researchers have focused on all aspects of PD-L1. Regarding the expression of PD-L1, some studies have shown that the higher the PD-L1 expression, the better the therapeutic effect of PD-L1 immune checkpoint drugs [7–9]. In fact, a phase 3 trial has suggested an association of increased tumoral PD-L1 expression with better clinical efficacy. In particular, KEYNOTE-024 showed that in advanced NSCLC patients with no *EGFR* mutations or no *ALK* fusion who showed high tumoral PD-L1 expression (≥50%), pembrolizumab seemed to be superior to platinum-based chemotherapy [10]. Additionally, KEYNOTE-010 showed that for advanced NSCLC with tumoral PD-L1 expression ≥1%, pembrolizumab was superior to docetaxel [11].

Many papers have researched factors related to the expression of PD-L1, but their conclusions are controversial. Skov BG et al. report that the expression of PD-L1 is only related to the clinical stage of non-small cell lung cancer, which suggests that a lower stage is associated with a lower prevalence of PD-L1 positivity [12], while Zhang et al. detected an association between high PD-L1 expression and male sex and smoking history [13]. To a certain extent, the reason for the dispute is that, on the one hand, the detection reagents are different, some are genetic testing at the mRNA level, while some are immunohistochemistry testing at the protein level, and there are currently three kinds of antibodies in the detection of protein level [14–16]. On the other hand, there are subjective differences in the interpretation of pathological doctors [17]. Therefore, the purpose of our study is to collect and analyze a large number of clinical samples, interpret the mechanism on the basis of a great number of studies in the literature, and come to a conclusion on the correlation between the expression of PD-L1 and the sex, age, histological type smoking history, tumor maximum diameter, TNM stage and clinical stage of patients. Our aim is to provide evidence for clinicians and patients when they are jointly formulating treatment plans as to whether anti-PD-L1 immunotherapy might be an option.

## Materials and Methods

### Patients characteristic

We established a database of patients who were diagnosed with non-small cell lung cancer in the pathology department of Xiangya Hospital from January 2016 to June 2021. The histological analysis was based on the WHO classification standard of lung tumors in 2015 [18]. In some of these cases, the tissue wax blocks were too small or with improper conservation so that there was no sufficient qualified tissue for further experiment, hence, we ended up with 1008 cases of lung cancer tissues which were collected for PD-L1 IHC detection. Next, we analyzed the correlation between PD-L1 protein expression and patients’ clinicopathological characteristics, including sex, age, lung cancer histological type, smoking history, maximum tumor diameter, level of differentiation, TNM stage, and clinical stage. The clinical stage was determined according to the TNM stage of lung cancer in the 8th edition of IASLC in 2017 [19]. In addition, we obtained 1,013 cases from the TCGA database, including 498 squamous cell carcinoma patients and 515 adenocarcinoma patients. The information about the gene expression or gene variants was collected, where *PD-L1, EGFR, PGFR, KRAS, BRAF, DDR2, ROS-1, RET* and *ALK* were included, which were the most common driven gene in NSCLC. And a heat map of the gene expression was drawn to see the difference of gene distribution between LUSC and LUAD. Approval to review, analyze, and publish the data in this study was given by the Ethics Board of Xiangya Hospital of Central South University (No. 202302031).

### Immunohistochemical staining

**Figure 1:**
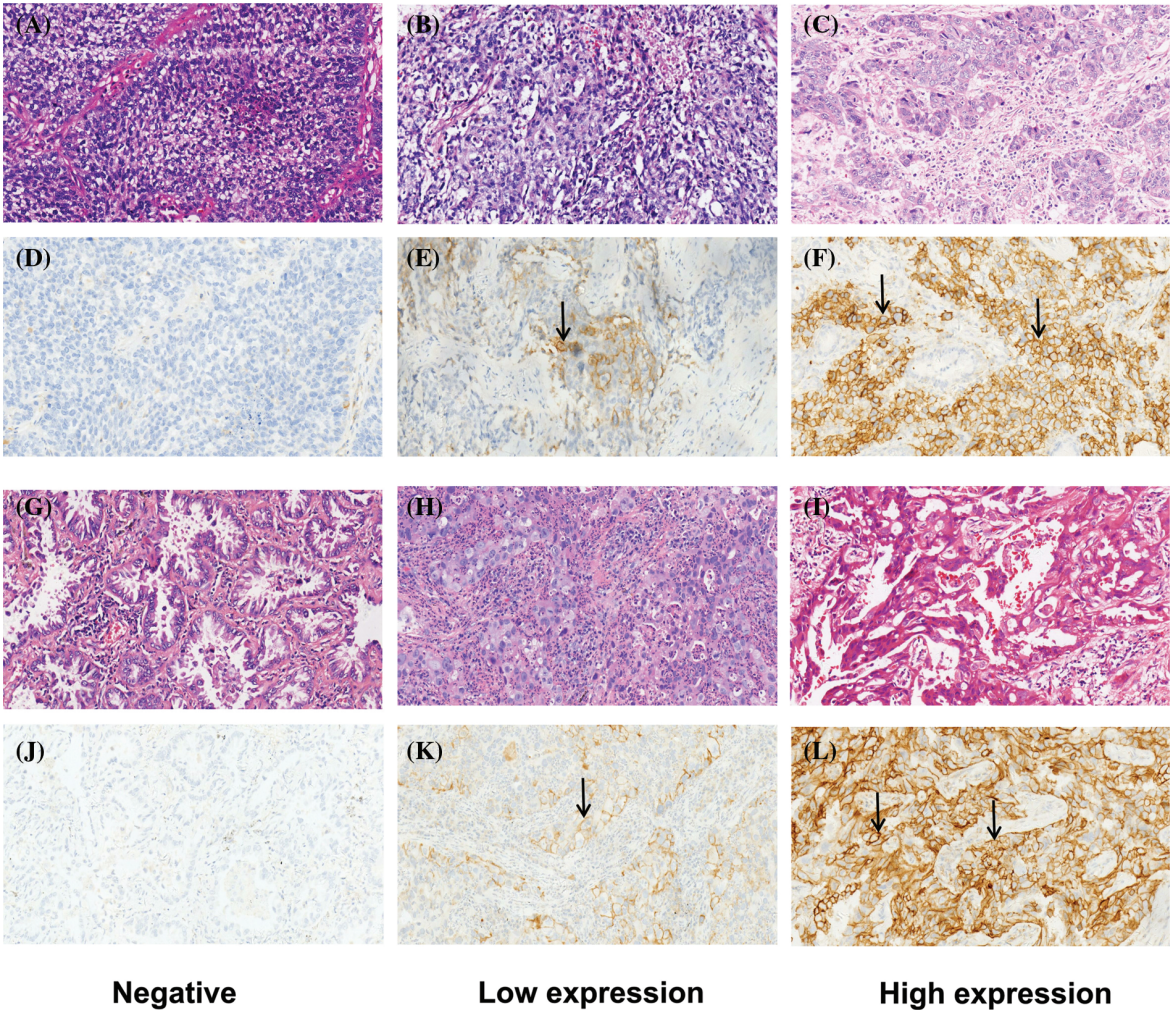
Typical patterns of HE staining and corresponding PD-L1 IHC staining (200X) about LUSC and LUAD. (A–F) are about LUSCs. (A–C) are HE staining; (D–F) are the corresponding PD-L1 IHC staining: (D) is negative (<1%), (E) is low expression (1%~49%), and (F) is high expression (≥50%). (G–L) are about LUADs. (G–I) are HE staining; (J–L) are the corresponding PD-L1 staining: (J) is negative, (K) is low expression, and (L) is high expression.

Immunohistochemistry staining of PD-L1 (PD-L1 IHC 22c3 pharmDx) was performed on Dako Autostainer Link 48 (Dako North American, Inc., Carpinteria, USA). Specimens were cut into 4 μm slices, mounted on a slide (Trajan® series 3), and dried in an oven at 60°C for 60 min. Dewaxed with xylene and rehydrated with graded alcohol (absolute ethanol, 95% ethanol, 75 ethanol). Antigen recovery and inhibition of endogenous peroxidase activity were performed in accordance with the manufacturer’s instructions. According to FDA approval, Dako.22c3 was used to detect PD-L1, and when the tumor cell membrane was stained, the tumor cells were considered positive. When the cytoplasmic immune response occurred, the tumor cells were considered negative [14,20]. The expression of PD-L1 was determined by the tumor proportion score (TPS), which refers to the percentage of living tumor cells showing partial or complete membrane staining (Fig. 1). The specimens were divided into 3 types according to TPS: negative (<1%); low expression (1%–49%); and high expression (50%–100%) [21–24]. Each patient’s slide reading was completed by two qualified pathologists who did not know the origin diagnostic and followed the blind principle. Disagreements were resolved with the help of a third and senior pathologist. Pathologists interpreted the results of PD-L1 IHC by combining hematoxylin and eosin staining slides.

### Statistical methods

Statistical analysis was performed using SPSS 26.0 statistical software. After we obtained the information of PD-L1 IHC staining from 1008 clinical samples, the results were classified into three types: negative, low expression, and high expression and assigned values (0,1,2) in statistical analysis. Chi-square tests were conducted to analyze whether PD-L1 expression was different among different groups (e.g., sex, age), *p* values < 0.05 were considered statistically significant. Spearman coefficient (ρ) from the correlation analysis was shows as an indication of the correlation between the independent and dependent variables (ρ value greater than zero means a positive correlation and less than zero means a negative correlation).

## Results

### PD-L1 expression in NSCLC patients

**Figure 2:**
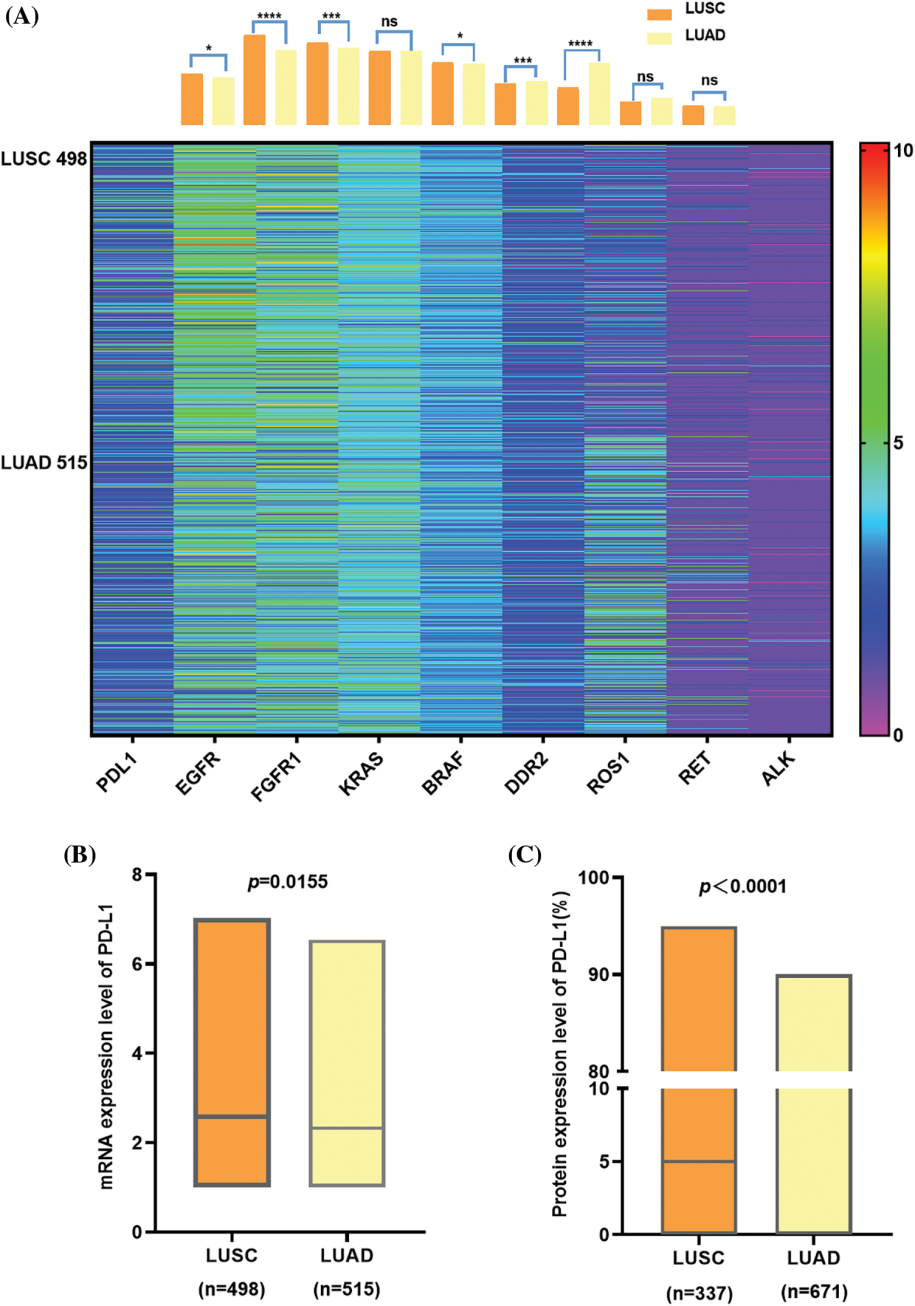
The comparison between LUSC and LUAD in lung cancer driver gene, PD-L1 expression at mRNA, and protein. (A) shows a heat map with a column bar of the lung cancer driver gene expression of NSCLC patients from the TCGA database. There is an apparent difference in the gene variation status of *EGFR, ROS-1* (*p* < 0.0001), *FGFR*, and *DDR2* (*p* < 0.001) between LUAD and LUSC, while on *KRAS*, *RET*, and *ALK*, obvious discrepancies do not exist. Regarding *PD-L1*, although the expression level is not very high in general, there are still divergences between the two subtypes. (B) At the mRNA level, *PD-L1* expression was higher in LUSC than in LUAD, and it was the same at the protein level (C) In addition, PD-L1 protein tends to be 0 in LUAD since the median was zero.

We obtained the gene expression information of NSCLC patients from the TCGA database and drew a heat map with column chart (Fig. 2A). There is an apparent difference in the gene variation status of EGFR, ROS-1 (*p* < 0.0001), FGFR and DDR2 (*p* < 0.001) between LUAD and LUSC, while on KRAS, RET and ALK, obvious discrepancies do not exist. Regarding PD-L1, although the expression level is not very high in general, there are still divergences between the two subtypes. Furthermore, we made a box plot of PD-L1 expression in LUADs and LUSCs to compare the differences. It was found that at mRNA level, PD-L1 expression was higher in LUSC than in LUAD (*p* = 0.0155) (Fig. 2B). As the same, PD-L1 protein seems to be higher in LUSC than LUAD (*p* < 0.0001) and compared to LUSC, PD-L1 is more likely to be close to 0 in LUAD (Fig. 2C).

### Correlation between PD-L1 protein expression and clinicopathologic characteristics of NSCLC patients

Overall, 1008 clinical samples were included in our study, and the patients’ relevant clinicopathological characteristics are shown in Table 1. Chi-square tests were conducted to analyze whether PD-L1 expression was different among different groups (e.g., sex, age). From univariate analysis, the expression of PD-L1 was significantly different from the patients’ sex, subtype, smoking history, maximum tumor diameter, differentiation degree, tumor (T), regional lymph node (N), metastasis (M), and clinical stage. The expression of PD-L1 was higher in patients who were male, had lung squamous cell carcinoma, a smoking history, a tumor diameter >3 cm, poor differentiation, and stages III~IV disease (*p* < 0.001). From multivariate analysis, the expression of PD-L1 was significantly different between lung cancer types and differentiation degrees. The expression of PD-L1 was higher in patients who had LUSC or poor differentiation (*p* < 0.001).

**Table 1:** Correlation between PD-L1 expression and NSCLC patients clinicopathologic characteristic

Variable	n (%)	PD-L1 TPS, n (%)			Univariate analysis		Multivariate analysis
		<1%	1%~49%	≥50%	*p* value	*ρ* value	*p* value
Sex (n = 1008)					0.000	−0.139**	0.497
Male	686(68.1)	345	217	124			
Female	322(31.9)	202	96	24			
Age, y (n = 1008)					0.644	0.015	0.873
≤65	713(70.7)	388	227	98			
M65	295(29.3)	159	86	50			
Histological type (n = 1008)					0.000	−0.168**	0.044
LUSCs	337(33.4)	148	114	75			
LUADs	671(66.6)	399	199	73			
Smoking history (n = 435)					0.031	0.103*	0.868
No	218(50.1)	136	64	18			
Yes	217(49.9)	120	56	41			
Tumor maximum diameter (n = 411)					0.000	0.213**	0.708
≤3	224(54.5)	157	53	14			
M3	187(45.5)	97	52	38			
Level of differentiation (n = 893)					0.000	0.191**	0.003
Well~ medium	282(31.6)	119	98	65			
Poor	611(68.4)	370	176	65			
Tumor(T) (n = 408)					0.000	0.240**	0.172
T1	208(51.0)	114	52	12			
T2	94(23.0)	59	21	14			
T3	61(15.0)	38	13	10			
T4	45(11.0)	11	19	15			
Region lymph node (N) (n = 408)					0.000	0.329**	0.089
N0	269(65.9)	192	63	14			
N1	29(7.1)	14	9	6			
N2	63(15.4)	32	17	14			
N3	47(11.5)	14	16	17			
Metastasis(M) (n = 408)					0.000	0.255**	0.351
M0	363(89.0)	240	84	39			
M1	45(11.0)	12	21	12			
Clinical stage (n = 408)					0.000	0.328**	0.148
I~II	282(69.1)	201	65	16			
III~IV	126(30.9)	51	40	35			

Note: ρ refers to Spearman coefficient. ρ value greater than zero means a positive correlation and less than zero means a negative correlation.

### PD-L1 protein expression differs by subtype and degree of differentiation

In 337 LUSC patients, we found that PD-L1 expression was correlated with T stage, N stage, and clinical stage. In 671 LUAD patients, we found that PD-L1 expression was correlated with sex, smoking history, differentiation degree, maximum tumor diameter, tumor (T), regional lymph node (N) metastasis (M), and clinical stage (Table 2).

**Table 2:** Difference of PD-L1 protein expression in LUADs and LUSCs

Histological type	Variable	*p* value	ρ value
LUSC			
	Tumor (T)	0.049	0.187*
	Region lymph node (N)	0.001	0.323**
	Clinical stage	0.003	0.278**
LUAD			
	Sex	0.003	−0.116**
	Smoking history	0.035	0.118*
	Differentiation	0.000	−0.200**
	Tumor maximum diameter	0.001	0.195*
	Tumor (T)	0.002	0.184**
	Region lymph node (N)	0.000	0.337**
	Metastasis (M)	0.000	0.324**
	Clinical stage	0.000	0.361**

Note: ρ refers to Spearman coefficient. ρ value greater than zero means a positive correlation and less than zero means a negative correlation.

In 282 cases of patients with well~medium differentiation (LUSCs or LUADs), we found that PD-L1 expression was correlated with age, sex, histological type, tumor maximum diameter, tumor (T), regional lymph node (N), metastasis (M), and clinical stage. In 611 patients with poor differentiation (LUSCs or LUADs), we found that PD-L1 expression was correlated with age, tumor (T) and regional lymph node (N) status (Table 3).

**Table 3:** Difference of PD-L1 protein expression in different differentiation groups

Differentiation	Variable	*p* value	ρ value
Well~ medium			
	Age	0.045	−0.081*
	Sex	0.000	−0.158**
	Histological type	0.000	−0.226**
	Tumor maximum diameter	0.010	0.146*
	Tumor (T)	0.027	0.127*
	Region lymph node (N)	0.000	0.280**
	Metastasis (M)	0.000	0.246**
	Clinical stage	0.000	0.306**
Poor			
	Age	0.017	0.142*
	Tumor (T)	0.048	0.200*
	Region lymph node (N)	0.029	0.219*

Note: ρ refers to Spearman coefficient. ρ value greater than zero means a positive correlation and less than zero means a negative correlation.

**Figure 3:**
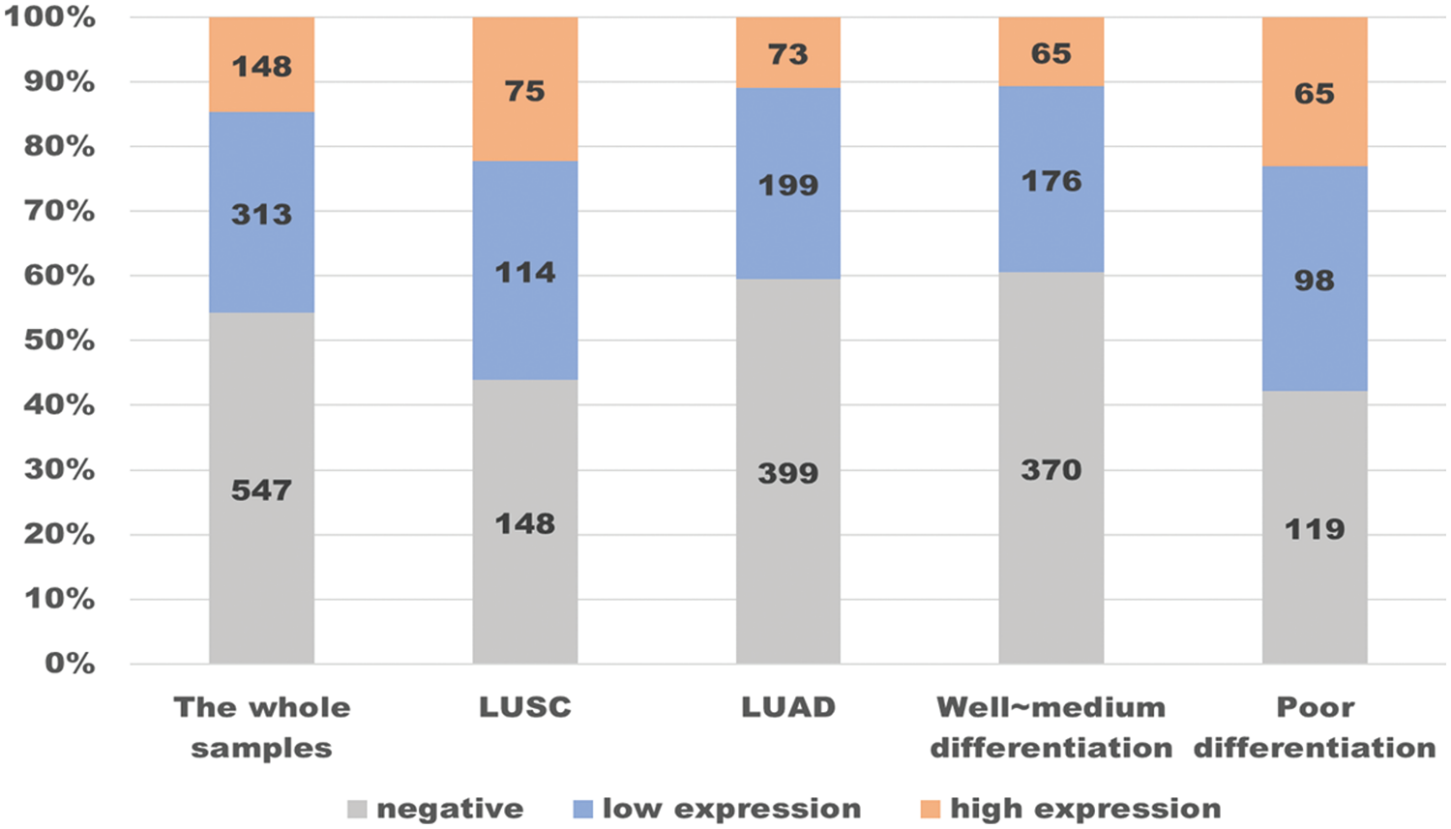
Distribution of PD-L1 expression in 1008 lung cancer samples.

The prevalence of PD-L1 expression is demonstrated in Fig. 3, where we can see the distribution. Among the 1,008 patients, 14.7% had a TPS ≥50%, 31.1% had a TPS 1% to 49%, and 54.3% had a TPS <1%. In particular, we conducted stratified analysis on LUSCs and LUADs or populations with poor differentiation and well to medium differentiation, and calculated the corresponding negative expression and high expression rates, as well as the 95% confidence interval (Table 4). In a well to medium differentiated adenocarcinoma patient in the early clinical stages (I~II) or with no regional lymph node metastasis, the possibility of PD-L1 negative expression was 78.4%. In an LUSC patient with lymph node metastasis (N1~N3), the probability of high PD-L1 expression was higher (R2 = 0.375, 95% CI 21.8%–53.2%). For a patient with a smoking history in the N3 stage, the probability of high PD-L1 expression was as high as 62.5% (95% CI 35.9%–89.1%).

**Table 4:** Summary of groups with high negative-rate(R0) or high-expression rate(R2)

Classification	PD-L1 TPS, n (%)					Sum	R0(95% CI)	R2(95% CI)
	<1%		1%~49%		≥50%			
Well~ medium differentiation &Clinical stages I~II &LUADs	145	36		4		185	0.784(0.724–0.884)	0.022(0.000–0.430)
Well~ medium differentiation &LUADs &N0	138	36		5		179	0.771(0.709–0.833)	0.028(0.004–0.052)
No smoking &LUADs &M0	113	33		4		150	0.753(0.684–0.823)	0.027(0.001–0.053)
Well~ medium differentiation &N0 &M0	173	44		7		224	0.772(0.717–0.828)	0.031(0.008–0.054)
Well~ medium differentiation &Clinical stages I~II	184	48		10		242	0.760(0.706–0.814)	0.041(0.016–0.067)
N1~N3 & LUSC	15	10		15		40	0.375(0.218–0.532)	0.375(0.218–0.532)
Poor differentiation &N3	7	9		13		29	0.241(0.076–0.407)	0.448(0.256–0.641)
Smoking &N3 &M0	3	3		10		16	0.188(0.040–0.460)	0.625(0.359–0.891)

Note: R0 means the rate of being negative in PD-L1 detection, R2 means the rate of being high expression in PD-L1 detection.

In the whole samples, almost half (547 in 1008) of PD-L1 expression was negative, while the proportion of high expression was 14.7%. By contrast, in patients with LUSC, the rate of PD-L1 ≥50% is higher than in the whole samples, while the rate of being negative is descending, as is the patients in poor differentiation. In contrast, in patients with LUAD or in well~ medium differentiation, the change in the rate of being negative and being high are completely opposite.

### Pathological characteristics of patients with high expression of PD-L1 protein

**Figure 4:**
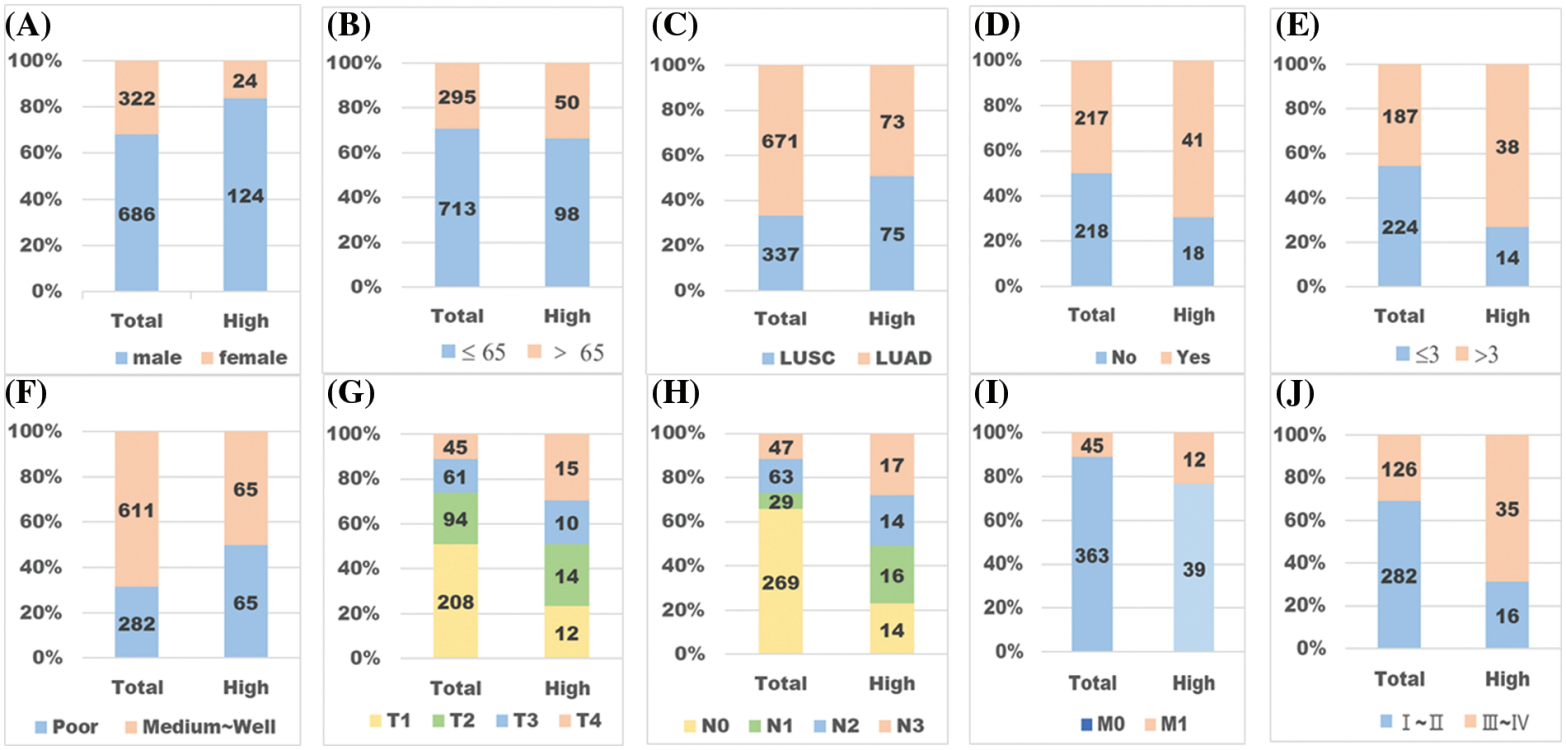
Pathological characteristics of patients with high expression of PD-L1. “Total” refers to the whole sample, and “high” refers to the high expression group. (A) Male or female. (B) Age <65 or age ≥65. (C) LUSC or LUAD. (D) NO smoking or smoking. (E) Tumor maximum diameter ≤3 cm or M3 cm. (F) Differentiation state: well~ medium or poor. (G) T stage. (H) N stage. (I) M stage. (J) Clinical stage. By comparing patients’ different pathological characteristics between the whole samples and the high expression group, we found some significant differences (*p* < 0.01). The one expressing PD-L1 highly is more likely to be a patient who is male (A), over 65 years old (B), LUSC (C), a smoker (D), with tumor diameter M3 cm (E), in poor differentiation (F), with a higher TNM stage (G~I), and in clinical stages III~IV (J).

We want to screen out the population with high expression of PD-L1 protein, which is the purpose of our study. Therefore, we summarized all the characteristics of the high expression population. Compared with the total 1,008 cases, the high expression group shows something different (Fig. 4). NSCLC patients with PD-L1 high expression are more likely to be male, over 65 years old, LUSC, smoker, tumor maximum diameter >3 cm, poor differentiation, T1–T3 stage, regional lymph node metastasis, tumor metastasis or clinical stages III–IV.

## Discussion

### PD-L1 protein expression in NSCLC

The most comprehensive study regarding PD-L1 protein expression was the EXPRESS study, which included 2,435 patients and evaluated PD-L1 IHC staining in samples obtained from local laboratories located in 45 centers in 18 countries. The percentages of patients with PD-L1 TPS ≥50% and TPS ≥1% were 22% *vs*. 51% in Europe, 22% *vs*. 53% in the Asia-Pacific region, 22% *vs*. 47% in the Americas, and 24% *vs*. 54% in other countries [25]. In our study, the ratio is 14.7% *vs*. 54.3%. The prevalence of PD-L1 was similar across geographic regions and broadly consistent with central testing results from a clinical trial screening population [25]; Naturally, our results are similar..

In 2016, Aggarwal et al. found a larger proportion of patients with TPS <1% in the subgroup with non-squamous cell carcinoma compared with squamous cell carcinoma (26% *vs*. 19%); that is, in LUADs, patients’ PD-L1 is more likely to be negative compared with LUSCs [26], which is similar to our findings. In our study, 59.46% of LUADs expressed PD-L1 negatively, while 43.91% of LUSCs expressed PD-L1 negatively. Because PD-L1 is increasingly familiar and recognized by the medical community and testing is affordable, a growing number of patients can access PD-L1 detection. Therefore, with the increase in detection rates, the overall negative rate has also increased, which is understandable. Similarly, in 2017, an association was observed between adenocarcinoma and low PD-L1 expression in NSCLC samples, including Chinese, Japanese, Korean, American, Canadian, British, and French individuals [12]. Additionally, the results indicated that PD-L1 expression was also associated with sex, smoking status, histology, differentiation, tumor size, lymph node metastasis, TNM stage, and *EGFR* mutation [12]. Moreover, this is one of the few studies that is consistent with our conclusion. However, Chen et al. concluded that a significant difference in PD-L1 expression between LUSCs and LUADs was observed, but in contrast, age, sex, and smoking history were not statistically significant [27]. Gelatti et al. were the first to comprehensively describe PD-L1 expression in the Brazilian population and a significant association between the histological subtype and the expression of PD-L1 was observed [28]. In China, a study by Song et al. showed that PD-L1 expression was associated with advanced stage, lymph node (LN) metastasis, solid predominant subtype and wild-type epidermal *EGFR* gene expression [29].

Taken together, there are some divergences in certain aspects between these studies of patients in various regions. However, this may be due to differences in the regional population, sample size, PD-L1 detection reagent, and PD-L1 expression interpretation threshold [17]. Therefore, we should try to find other experiments with small differences for analogy analysis. Despite the differences, a relatively unified opinion is that the expression of PD-L1 is related to the histological subtype of lung cancer. Generally, the expression of PD-L1 in LUADs is more likely to be lower than that in LUSCs, which is consistent with our research results.

### Statistical differences mainly exist in subtypes and differentiation degree

Until now, no general pathway to control PD-L1 expression has been discovered. Depending on stimulus and cell type, the expression of PD-L1 was found to connect with various signaling molecules: MAPKs [30], STAT family [31,32], IRF-1 [33], PIK3 [34] and etc. Studies have shown that PD-L1 is more highly expressed in squamous carcinoma, both at the protein level and at the mRNA level. So is it because there is a certain PD-L1-related signaling pathway that is more active in squamous carcinoma? We found that STAT3 in the STAT family is associated with PD-L1. Also discovered was that STAT3 in LUSC tissues showed increased expression level rather than in LUAD in the Talbot database [35]. Intercellular communication between tumor cells, immune cells, and the stroma characterizes the tumor microenvironment [36]. On the one hand, accumulating data have established that STAT3 directly regulates the expression of PD-L1 [37,38]; on the other hand, recent findings have shown that STAT3 represses CD8+ T cell chemotaxis and activation [39]. Additionally, PD-L1 expression has bearing on CD8 T cell infiltration within the squamous cell carcinoma microenvironment [40,41]. Based on these studies, many experiments on the expression of PD-L1, T cells, and STAT3 in squamous cell carcinoma have been carried out. Vasquez et al. obtained tumor-associated MDSCs derived from patients with head and neck squamous cell carcinoma, and it was found that pSTAT3 was at higher levels of expression to and suppressed T cell proliferation through the actions of arginase 1 [42]. Another trial conducted in oral squamous carcinoma cells by Xia revealed that pSTAT3 levels are elevated in CAFs cocultured with oral squamous carcinoma cells [43]. According to these studies, activation of the STAT3 pathway directly upregulates the expression of PD-L1. However, in different lung cancer subtypes, the expression of STAT family members is different. Hence the influence of the STAT family on the expression of PD-L1 in lung cancer and adenocarcinoma is worthy of further discussion.

Consistent with most studies, we concluded that the expression of PD-L1 is related to the type of NSCLC. However, we also believe that a significant difference exists in the differentiation states of lung cancer tissues. We know that cellular differentiation, or simply cell differentiation, is the process through which a cell undergoes changes in gene expression to become a more specific type of cell. These changes are largely due to highly controlled modifications in gene expression and are the study of epigenetics [44–47]. In other words, during the process of cell differentiation, cell morphology tends to exhibit certain histology features, which is the fundamental basis for the microscopic diagnosis of lung squamous cell carcinoma or lung adenocarcinoma. In our study, many statistical results showed that tumor differentiation is related to PD-L1 expression; in particular, a patient with well to medium differentiation, has a 76.0% to 78.4% probability of having a negative PD-L1 expression. Although findings in other studies differ, there are subjective distinctions in the judgment of differentiation in various studies. It is worth noting that our research sample is as large as possible to reduce this error, so we are more confident in our conclusions. Because there is no research on the molecular mechanism as theoretical support for this controversy, more work needs to be conducted.

In our univariate analysis, we also found that PD-L1 expression at protein level was significantly different in smoking, sex, and lymph node metastasis. It was found that the efficacy of pembrolizumab, an antibody is correlated with the molecular smoking signature and a better ORR of immunotherapy was found in tobacco smoking patients than no smokers. [48–50]. Smoking status may be a predictive marker for better survival as PD-1/PD-L1 inhibitors [51] and total smoking duration may be a predictor of a PD-L1 TPS ≥50% (*p* = 0.001) [52], which is consistent with our study. In our study, nearly half of the patients were smokers, and there was a significant difference in their PD-L1 expression (*p* = 0.031). We all know that in China, the proportion of male smokers is much larger than that of female smokers, and our data also show the same (the correlation analysis between smoking history and sex: *p* = 0.015). So, to some extent, it is comprehensible when smoking history and sex are factors related to the expression of PD-L1. However, gender differences themselves have an impact on the diseases [53–55]. Preclinical studies suggest that the expression of PD-L1 is modulated in an estrogen-dependent and sex-dependent manner [53,54]. Sex-related differences in the anticancer immune response have been described in tumor expression levels of PD-L1 across a large spectrum of tumors, including NSCLC [56–58]. This difference in lung cancer treatment based on gender differences also verifies our experimental results and affirms the significance of our study. In our research, there were 99 female patients who were medium or well differentiated with no lymph node metastasis; their negative expression rate was as high as 78.8% (95% CI: 70.6%–87.0%).

Lymph node metastasis is the most common method of metastasis of lung cancer [59–61]. Recent studies have demonstrated that the expression of PD-L1 may be mediated by the PI3K/AKT/PTEN pathway [62–64]. The activated PI3K-AKT pathway plays a central role in the translation of interferon-γ, which is a key regulator of PD-L1 transcription [34,64]. Up to 50% of penile cancers positively express PD-L1, and PD-L1 is positively related to LNM [65,66]. In addition, Hu et al. first showed a positive linear correlation between NLR and PD-L1 in penile cancer. Such a linear correlation was also reported in cholangiocarcinoma [67]. These conclusions combined with our experimental results show that there is a relationship between PD-L1 expression and lymph node metastasis. Although the specific mechanism is not completely clear, it also provides guidance for future research. In our study, the expression of PD-L1 in patients without lymph node metastasis was usually negative or low. In contrast, while a smoker patient was in the N3 stage with no distant metastasis, he showed high expression of PD-L1 (R2 = 62.5%, 95% CI: 35.9%–89.1%).

## Conclusion

By analyzing the correlation between the expression level of PD-L1 and clinicopathological parameters in 1,008 LUSCs or LUADs, we conclude that the expression of PD-L1 seems to be higher in males, smokers, squamous cell carcinoma tumors with a maximum diameter >3 cm, patients with poor differentiation and/or high TNM stage. Among patients with well- to moderate-differentiated lung adenocarcinoma, clinical stages I~II, with no smoking history, lymph node metastasis or distant metastasis, we would not routinely recommend PD-L1 testing. However, we strongly recommend testing for patients with LUSCs who have lymph node metastasis or poorly differentiated NSCLC in N3, especially smokers in the N3 stage. If high PD-L1 expression is revealed, it will be effective in guiding targeted therapy. Naturally, other categories that are not mentioned should be considered according to the needs of the patient.

Compared with similar studies, our study has a larger sample size. At the same time, we have more detailed groups of patients with the goal of trying to determine which patient populations have a high expression PD-L1 high expression, the focus of our study. However, there are limitations worth noting. We cannot analyze the relationship between PD-L1 expression and prognosis. We also selected cases from 2016 to the present, and the time span causes deviation in clinical information. In the future, our team will continue to study lung cancer-related molecules and hope to compensate for the deficiencies in this study.

## Data Availability

The datasets used and/or analyzed during the current study are available from the corresponding author on reasonable request.

## Abbreviation

HE: Hematoxylin and eosin
IHC: Immunohistochemical
SCLC: Small cell lung cancer
NSCLC: Non-small cell lung cancer
LUAD: Lung adenocarcinoma
LUSC: Lung squamous cell carcinoma
LCC: Large cell cancer
FDA: Food and Drug Administration
PD-L1: Programmed cell death protein 1 ligand 1
EGFR: Epidermal growth factor receptor
ALK: Anaplastic lymphoma kinase
PD-1: Programmed cell death protein 1
TNM stage: Tumor, Region lymph node, Metastasis stage
WHO: World Health Organization
IASLC: International Association for the Study of Lung Cancer
TCGA: The Cancer Genome Atlas
TPS: The tumor proportion score
LN: Lymph node
